# Reversible cerebral vasoconstriction syndrome with basilar artery stenosis: A case report

**DOI:** 10.1097/MD.0000000000027337

**Published:** 2021-09-24

**Authors:** Sang Woo Joh, Sang Yeon Kim, Byoung-Soo Shin, Hyun Goo Kang

**Affiliations:** a Jeonbuk National University Medical School, Jeonju, South Korea; b Department of Neurology, Jeonbuk National University Medical School and Hospital, Jeonju, South Korea; c Department of Neurology, Research Institute of Clinical Medicine of Jeonbuk National University - Biomedical Research Institute of Jeonbuk National University Hospital, Jeonju, South Korea.

**Keywords:** basilar artery, headache, posterior circulation ischemia, reversible cerebral vasoconstriction syndrome

## Abstract

**Introduction:**

Acute severe headaches in young patients may be associated with fatal neurological complications that necessitate imaging examinations. Among acute severe headaches, a thunderclap headache may indicate the rupture of a cerebral aneurysm or the onset of reversible cerebral vasoconstriction syndrome for which emergent evaluation is required.

**Patient concerns:**

We report the case of a 36-year-old man who presented to our hospital with an acute severe headache after excessive exercise the previous day. He was prescribed a pain reliever and discharged under the suspicion of vestibular migraine but returned to the emergency room after 4 hours due to right hemiparesis, right facial palsy, severe dysarthria, and a mild drowsy mental status.

**Diagnosis:**

After cerebral angiography, we diagnosed basilar artery stenosis with acute infarction in the posterior circulation due to reversible cerebral vasoconstriction syndrome.

**Interventions:**

Brain computed tomography angiography revealed complete occlusion of the vertebrobasilar artery. Transfemoral cerebral angiography showed spontaneous improvement in the occlusion before thrombectomy.

**Outcomes:**

Ten months later, high-resolution vessel wall magnetic resonance angiography showed persisting severe stenosis of the basilar artery.

**Conclusions:**

A headache in young patients with risk factors of atherosclerosis, such as smoking history, uncontrolled hypertension, and dyslipidemia may be caused by reversible cerebral vasoconstriction syndrome or ischemic stroke, which has fatal neurological complications. Therefore, reversible cerebral vasoconstriction syndrome or ischemic stroke should be suspected and appropriately evaluated in such patients, even if the headache is not the thunderclap type.

## 1 Introduction

Almost everybody experiences headaches, most of which are benign primary headaches such as migraine, tension headaches, and cluster headaches.^[1]^ While an estimated 4% of all headaches are secondary headaches, they can be caused by life-threatening conditions.^[2]^ Therefore, acute severe headaches warrant suspicions of being secondary headaches and the possible underlying diseases should be investigated.^[2]^ Among acute severe headaches, a thunderclap headache (TCH) may indicate the rupture of a cerebral aneurysm or the onset of reversible cerebral vasoconstriction syndrome (RCVS), which requires emergent evaluation.^[2]^ RCVS is characterized by repeated acute TCHs and diffuse segmental constriction in the cerebral artery, which naturally improves within 3 months from onset, and is accompanied by neurological complications.^[3–5]^ RCVS can occur in the circle of Willis and its adjacent branches. It occurs most frequently in the middle cerebral artery but can also occur in blood vessels of the posterior cerebral circulation, leading to the development of various neurological symptoms.^[6,7]^

A young man visited our hospital for a headache that was initially thought to be a vestibular migraine. Neurological symptoms corresponding to posterior circulation ischemia (PCI), angiography findings characteristic of RCVS, and acute infarction in the brainstem were observed. Herein, we report this case along with a literature review.

## 2 Case report

A 36-year-old male patient visited our hospital for an acute headache that had occurred after exercising excessively the previous day. He reported that he had experienced a headache accompanied by blurred vision several times in the past. The headache was right-sided, pulsatile, and pounding, with a numerical rating scale score of 8, and was accompanied by vertigo (spinning-type) and vomiting. The patient had a history of 12 pack-years of cigarettes and had been diagnosed with hypertension but was not taking any medication. His blood pressure was high (163/103 mmHg) at the time of visit; however, there were no other abnormal findings. All neurological examination results were normal. The patient was thought to have a vestibular migraine; therefore, magnetic resonance imaging (MRI) was planned to identify other brain lesions and he was discharged with a naproxen prescription.

**Figure 1:**
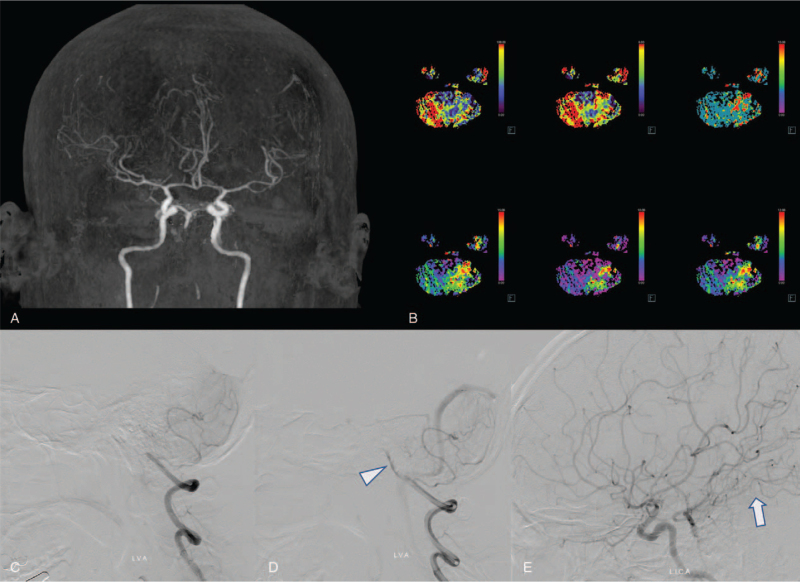
Brain CT angiography showing complete occlusion of the proximal portion of the vertebrobasilar artery (A). Perfusion CT showing a perfusion anomaly at the left cerebellum (B). Initial transfemoral cerebral angiography showing complete occlusion of the vertebrobasilar artery with thrombus filling (C). Spontaneous recanalization of the occluded vessel without thrombectomy (D, arrowhead). Follow-up angiography showing good blood supply in the posterior circulation, indicating a good collateral flow of the distal portion of the basilar artery (E, arrow). CT = computed tomography.

The patient returned to the emergency room 6 hours after he was discharged due to right-sided weakness that had occurred 4 hours after discharge. While he was initially able to write letters, it worsened over time and severe dysarthria was accompanied by the headache. Neurological examination results showed slight drowsiness. Right facial palsy was observed, and right hemiparesis was confirmed as Medical Research Council grade I/II. His National Institutes of Health Stroke Scale score was 11. Complete occlusion of the vertebrobasilar artery was observed on immediate brain computed tomography (CT) angiography (Fig. 1A). A perfusion anomaly of the posterior circulation was also detected by brain perfusion CT (Fig. 1B). Therefore, transfemoral cerebral angiography (TFCA) was conducted immediately for intra-arterial thrombectomy after administering an intravenous tissue plasminogen activator based on the evaluation that it was a symptom of acute occlusion. Upon initiation of TFCA, complete occlusion of the basilar artery was observed; however, the flow in the basilar artery gradually improved without a thrombectomy. The collateral flow at the distal portion of the basilar artery was good and the diameter of the existing basilar artery was very narrow (Fig. 1C-E).

**Figure 2:**
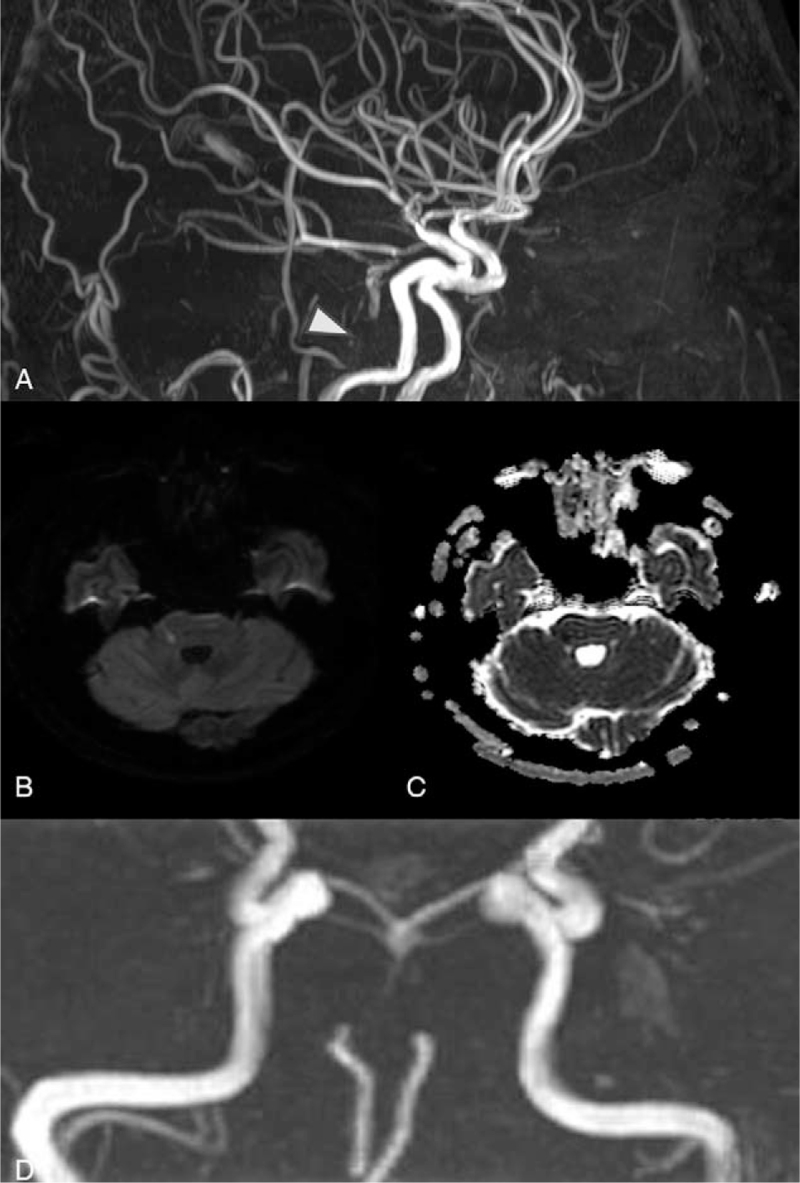
Brain MRA was conducted the next day after symptom onset, showing complete occlusion of the proximal portion (arrowhead) and good collateral flow through the distal portion of the vertebrobasilar artery (A). Focal infarction of the right lateral pons is observed in a diffusion-weighted image (B) and based on apparent diffusion coefficient (C). Persistent and severe luminal narrowing of the basilar artery on high-resolution vessel wall MRA was observed 10 months after symptom onset (D). MRA = magnetic resonance angiography.

Following this, the patient's neurological symptoms improved, he became mentally alert, his facial palsy and dysarthria improved, and a right motor Medical Research Council grade of IV/IV was observed. Brain MRI performed the next day showed acute infarction of the right lateral pons with chronic stenosis in the proximal portion of the vertebrobasilar artery and good collateral flow in the distal portion of the basilar artery (Fig. 2A-C). Considering the patient's age and blood vessel condition, evaluation for young age-related stroke risk factors was performed but did not reveal any specific findings other than increased low-density lipoprotein cholesterol concentration (179 mg/dL). All symptoms were completely relieved within 1 week with a dual antiplatelet agent (aspirin and clopidogrel) and statin (atorvastatin 20 mg) treatment, which were initiated the day after symptoms appeared. High-resolution vessel wall MR angiography performed after 10 months showed persisting severe stenosis of the basilar artery (Fig. 2D).

## 3 Discussion

A young man with a history of smoking, uncontrolled hypertension, and dyslipidemia experienced a headache after intensive exercise. Although the headache pattern met the diagnostic criteria for vestibular migraine, acute abnormal neurological symptoms were observed several hours later, and basilar artery occlusion was confirmed. TFCA showed slow and spontaneous recovery of the basilar artery flow without any special treatment. Even though the stenosis of the basilar artery persisted, all of the patient's symptoms improved. We suspected that the patient, given the chronic basilar artery stenosis, had developed RCVS, as the neurological symptoms occurred with an acute headache. The diameter of the basilar artery was narrow and the collateral flow at the distal portion was good. The basilar artery occlusion improved slowly over time; however, severe stenosis of the basilar artery persisted, as detected by high-resolution vessel wall MR angiography performed after 10 months.

RCVS is characterized by an acute severe headache and diffuse segmental cerebral vasoconstriction.^[3]^ It can sometimes be accompanied by sudden neurological symptoms.^[3]^ While recurrent TCHs are the most common symptom of RCVS and have a typical pattern, they are not essential for diagnosing RCVS.^[8]^ RCVS may manifest without a TCH, which is non-typical; moreover, some cases of RCVS do not present any headache.^[8]^ Because our patient did not present with a TCH at his first visit, it was difficult to immediately suspect RCVS. Unlike TCH, which is characterized by a hyperacute onset with intense pain reaching the highest intensity within 1 min and lasting approximately 1 to 3 hours, RCVS without TCH may be characterized by various developmental courses and pounding pain lasting from several minutes to several days and at various intensities.^[3,4,8]^ The most common neurological symptom associated with RCVS without TCH, one-sided motor deficits, may be accompanied by hemianopia, aphasia, or declining mental status.^[8]^ Our patient reported the rapid development of a unilateral pounding headache after 2 days of excessive exercise, which was accompanied by neurological complications such as vomiting, hemiparesis, and declining mental status. The occluded basilar artery, confirmed with TFCA, was slowly and spontaneously recanalized. These findings were similar to those in RCVS without TCH.^[3,4,8]^

The headache in our patient could also be attributed to PCI. The risk of a headache caused by an ischemic stroke is higher in the posterior circulation owing to its denser trigeminovascular innervation than that of the anterior circulation.^[9–11]^ Moreover, since the posterior circulation supplies blood to the brainstem, cerebellum, and occipital cortex, a headache may occur with related symptoms.^[11,12]^ A headache induced by an ischemic stroke may occur before, after, or during a stroke but most commonly occurs on the day of stroke symptom onset and can last from several days to several years.^[9,10]^ These headache patterns are related to the features of tension headaches and present with pressure-like or throbbing pain.^[9,10]^ Although the intensity is mostly mild to moderate, they are more severe when PCI occurs.^[9,10]^ The headache in our patient may have been caused by an ischemic stroke as his basilar artery stenosis was associated with the blood supply to the posterior circulation and his headache was pounding and severe. It also lasted for 2 days, along with focal weakness, facial palsy, signs of dysarthria, vertigo, vomiting, and disturbed consciousness that are characteristic of PCI. Acute infarction of the brainstem was also detected on MRI.^[11,12]^ However, it was more likely that the headache was caused by RCVS, as the TFCA findings showed spontaneous recanalization of the occluded basilar artery.

The patient in this case likely developed ischemia due to RCVS in the basilar artery, which had chronic stenosis. Due to the chronic stenosis, it was more easily occluded than the other blood vessels. Such chronic stenosis may be related to risk factors associated with atherosclerosis, such as smoking, hyperlipidemia, and long-term uncontrolled hypertension.^[13]^ A headache in a young patient with risk factors for atherosclerosis such as smoking history, hypertension, and hyperlipidemia, may be caused by RCVS or ischemic stroke, which can lead to fatal neurological complications. This should be considered during diagnosis. In the case of RCVS, the use of drugs, such as a triptan without imaging to ascertain the headache cause may be dangerous because it can cause vasoconstriction. Therefore, treatment should be administered after a comprehensive assessment of symptoms.

## Acknowledgments

We thank Editage (www.editage.co.kr) for English language editing.

## Author contributions

BSS, HGK, and SWJ were responsible for the research design. SYK, BSS, and SWJ performed data collection and analysis. HGK and SWJ performed the computational studies and wrote the manuscript. All authors have read and approved the final manuscript.

**Conceptualization:** Sang Woo Joh, Byoung-Soo Shin, Hyun Goo Kang.

**Data curation:** Hyun Goo Kang.

**Funding acquisition:** Hyun Goo Kang.

**Investigation:** Sang Woo Joh, Sang Yeon Kim.

**Methodology:** Sang Woo Joh, Sang Yeon Kim, Byoung-Soo Shin, Hyun Goo Kang.

**Supervision:** Byoung-Soo Shin, Hyun Goo Kang.

**Validation:** Sang Yeon Kim, Hyun Goo Kang.

**Visualization:** Sang Yeon Kim, Byoung-Soo Shin.

**Writing – original draft:** Sang Woo Joh.

**Writing – review & editing:** Byoung-Soo Shin, Hyun Goo Kang.
